# In Silico Characterization and Gene Expression Analysis of Toll Signaling Pathway-Related Genes in *Diaphorina citri*

**DOI:** 10.3390/insects13090783

**Published:** 2022-08-29

**Authors:** Mahnaz Rashidi, Nabil Killiny

**Affiliations:** Citrus Research and Education Center and Department of Plant Pathology, University of Florida, 700 Experiment Station Rd., Lake Alfred, FL 33850, USA

**Keywords:** Asian citrus psyllid, *Diaphorina citri*, Toll system, Huanglongbing, gene expression

## Abstract

**Simple Summary:**

*Diaphorina citri*, the vector of the citrus greening disease pathogen ‘*Candidatus* Liberibacter asiaticus’, has a reduced immune system in comparison to the non-hemipteran insects, including those of Lepidoptera, Dipterans, and Hymenoptera. Other hemipterans, such as *Acyrthosiphon pisum*, and *Rhodnius prolixus*, possess a partial or absent immune deficiency (IMD) pathway. *D. citri* does not have the IMD pathway, but has a complete Toll system that protects them from Gram-positive bacteria. Here we characterized the Toll signaling system genes in *D. citri*. In addition, we studied the expression profile of Toll genes in different life stages. Discovering this system and its dynamic expression may help in designing an innovative strategy by which we can control this insect vector.

**Abstract:**

The Asian citrus psyllid, *Diaphorina citri* is the main vector of citrus greening disease, also known as Huanglongbing (HLB). Currently, mitigating HLB depends on the control of *D. citri* using insecticides. To design innovative control strategies, we should investigate various biological aspects of *D. citri* at the molecular level. Herein we explored the Toll signaling system-related proteins in *D. citri* using in silico analyzes. Additionally, the transcripts of the identified genes were determined in all life stages from eggs to adults. Our findings reveal that *D. citri* genome possesses Toll signaling pathway-related genes similar to the insect model, *Drosophila melanogaster*, with slight differences. These genes include *cact*, *TI*, *Myd88*, *Dif/DI*, *pll*, *tub*, and *spz* encoding Cactus, Toll, Myeloid differentiation factor 88, Dorsal related immunity factor/Dorsal, Pelle, Tube, and Spaetzle, respectively. Unlike *D. melanogaster*, in *D. citri* Dorsal, immunity factor and Dorsal are the same protein. In addition, in *D. citri*, Pelle protein possesses a kinase domain, which is absent in Pelle of *D. melanogaster.* Gene expression analysis showed the transcript for *cact*, *TI*, *Myd88*, *pll*, *tub*, and *spz* are maximum in adults, suggesting the immunity increases with maturity. Instead, *Dif/DI* transcripts were maximal in eggs and adults and minimal in nymphal stages, indicating its role in embryonic development. The overall findings will help in designing pioneering control strategies of *D. citri* based on repressing its immunity by RNAi or CRISPR and combining that with biological control.

## 1. Introduction

Citrus greening disease, also known as Huanglongbing (HLB), is one of the most destructive citrus diseases worldwide [1]. It threatens citrus production in countries of Africa, Asia, South America, and North America [2,3]. In the Americas, citrus greening disease was first reported in São Paulo state in Brazil in 2004 and Florida, USA in 2005 [1,4,5,6]. Afterwards, reports of HLB occurred in Louisiana (2008), Georgia, and South Carolina (2009), and in California and Texas (2012) in the United States [7]. Furthermore, HLB was reported in Mexico, Jamaica, Belize, Puerto Rico, and Cuba [7]. Citrus greening is responsible for losses of millions of dollars in citrus in the United States, and the sustainability of the USD 9.3 billion citrus industry was affected by HLB in Florida, the largest citrus-producing state in the United States [8]. The putative causal agent of HLB, ‘*Candidatus* Liberibacter asiaticus’ is a Gram-negative bacterium, not available in culture, and phloem-limited [1,9,10]. ‘*Ca.* L. asiaticus’ is transmitted by Asian citrus psyllid, *Diaphorina citri* Kuwayama (Hemiptera: Liviidae) in a circulative–propagative manner [1,3,11]. All commercial citrus cultivars, regardless of rootstocks, are susceptible to HLB [1]. Presently, no definitive curative treatments against HLB exist, and all control methods are failing to stop the worldwide increase in citrus greening disease [12]. Currently, management of HLB relies heavily on insecticidal control of the vector to decrease the population of *D. citri*, and thus, to reduce the spread of ‘*Ca.* L. asiaticus’ [1,13,14]. Application of insecticides is environmentally unfriendly and costly, and long-term insecticide use can negatively affect populations of natural enemies [15]. Additionally, the repeated use of insecticides could develop resistance in *D. citri* populations [16,17,18]. The application of broad-spectrum insecticides was demonstrated to be an efficient method for decreasing *D. citri* populations, especially if overwintering adult populations were targeted during tree dormancy [14]. In addition, average yields increased with the application of insecticides, but the increased cost of using insecticides may not be recovered [19,20,21]. Therefore, understanding the biology of vector–pathogen interactions is required to develop an eco-friendly and sustainable management strategy as an alternative to/or in combination with chemical controls. Additional information about insect immunity will help in designing innovative control strategies. Thus, studying the functional genomics of key pathways related to vector immune responses, such as the insect immune deficiency (IMD) and Toll signaling pathway is critical.

In general, insects lack adaptive immune systems [22], but they have powerful innate immunity that plays an important role in the defense against pathogens, and regulates the interactions with invading microorganisms [23,24,25,26,27,28]. The insect innate immune response contains humoral and cellular defense responses activated through the detection and binding of pathogen-associated molecular patterns (PAMs) from microbial surface structure to pattern recognition receptors (PRRs) [29,30,31,32]. In insects, various conserved signaling pathways activate immunity, including the Toll signaling pathway, the immune deficiency (IMD), Janus kinase/signal transducer and activator of transcription (JAK/STAT) pathway, and RNA interference (RNAi) [32,33,34,35,36,37]. The Toll and IMD pathways are NF-κB-related pathways and participate in a humoral immune response that functions through the production of antimicrobial peptides (AMPs) [22,33,38,39]. The Toll pathway is activated by fungi, many Gram-positive bacteria, and virus infection, whereas the IMD is triggered by Gram-negative bacteria but in a similar fashion to the Toll pathway [22,27,35,40,41]. The signaling pathways related to innate immunity of insects were studied in different insect species, particularly in Dipteran and Lepidopteran insects [26,42,43].

*D. citri* has a reduced immune system compared to the non-hemipteran insects including those of Lepidoptera, Diptera, and Hymenoptera [44,45,46,47]. Other hemipterans, such as *Acyrthosiphon pisum* Harris, *Rhodnius prolixus* Stal, and *Siphonaptera*, *Pediculus humanus* Linnaeus possess a partial or absent IMD pathway [48,49,50]. *D. citri* has no IMD pathway but it has a complete Toll system that improves its survival after infection with Gram-positive bacteria [47,51]. Because of the reduced immune system in *D. citri* and some other insects, such as *A. pisum*, the insects compensate this deficiency by using bacterial endosymbionts. It is reported that *Wolbachia* symbiont activate the immune system in mosquitoes and cause resistance against the pathogens [52]. ‘*Candidatus* Profftella armatura’ in *D. citri* produces a cytotoxic polyketide toxin, diaphorin, and act as a potential defensive symbiont against pathogens [53,54]. It was shown that diaphorin increases in ‘*Ca*. L. asiaticus’-infected *D. citri* [54]. Thus, our objective was to identify Toll signaling pathway-related genes in *D. citri* using the *Drosophila melanogaster* Toll pathway genes and protein sequences as inquiries in BLAST. We used bioinformatic techniques to identify Toll signaling pathway-related proteins and predict their three-dimensional structure, active domains, and binding-receptor profiles. We performed RT-PCR to study the gene expression profiles for all predicted Toll genes in *D. citri* developmental stages from egg to adult.

## 2. Materials and Methods

All experiments were performed at the laboratories and facilities of the Citrus Research and Education Center (CREC), Institute of Food and Agriculture Sciences (IFAS), University of Florida, Lake Alfred, Florida.

### 2.1. D. citri Colonies

The Asina citrus, psyllid, *D. citri*, was originally collected in 2014 from citrus groves in Polk County, Florida, USA. Colonies of *D. citri* were continuously reared on *Citrus macrophylla* and *Murraya koenigii* (curry leaf) in secured growth rooms (27 ± 2 °C, 60 ± 5% relative humidity, and 16:8 h L/D photocycle) at the Citrus Research and Education Center (CREC), University of Florida, Lake Alfred, Florida. The adults of *D. citri* and samples of *Citrus macrophylla* leaves were tested randomly every month for the presence of ‘*Ca*. L. asiaticus’ by polymerase chain reaction (PCR) assays described by Tatineni et al. (2008) [55] to keep colonies ‘*Ca*. L. asiaticus’-free.

### 2.2. Protein–Protein BLAST (BLASTp) Analysis

The protein–protein BLAST (BLASTp) algorithm from the basic local alignment search tool (BLAST) was performed to identify sequences of Toll signaling pathway-related genes in *D. citri* that resembled the query amino acid sequence of known Toll pathway genes of the model insect, *Drosophila melanogaster*. In brief, the protein sequences of Toll pathway-related genes Cactus (*Cact* gene; AAA85908.1) [56], Dorsal-related immunity factor (*Dif* gene; AAA28465.1) [57], Toll (*TI* gene; AAA28941.1) [56], Pelle (*Pll* gene; AAA28750.1) [58], Tube (*tub* gene; P22812.4) [59], Dorsal (*Di* gene; NP_724054.1) [57], Myeloid differentiation factor 88 also known as Myd88 (*Myd88* gene; AAL56570.1) [58], and Spätzle also known as Spaetzle (*Spz* NP_733194.1) [60], from *D. melanogaster,* were blasted against *D. citri* using protein–protein BLAST (BLASTp 2.11.0^+^) [61,62], based on recently available data on two major database websites, including the National Center for Biotechnology Information website (NCBI, https://www.ncbi.nlm.nih.gov/gene/, 15 April 2021), and “*Diaphorina citri* OGS v2.0 proteins” and “*Diaphorina citri* OGS v2.0 CDS” Blast database available in the Citrus Greening Solutions website (https://citrusgreening.org/organism/Diaphorina_citri/genome, accessed on 17 April 2021) [63], using compositionally adjusted substitution matrices [61]. Myd88 and Spaetzle proteins were not found using the BLASTp search in NCBI; therefore, we used the Citrus Greening Solutions website for the BLASTp search for those proteins. We created a short list of top matches based on more than 25% similarity with high coverage, and E-values close to zero, and excluded all partial, hypothetical, unknown, and low-quality proteins from the list. BLASTp revealed that the Dorsal-related immunity factor and Dorsal are the same protein in *D. citri.*

### 2.3. Sequence Alignment and Phylogenetic Tree

For each protein, top-matched amino acid (AA) sequences from different insect families showing high similarity with Tube, Pelle and Dorsal proteins from *D. melanogaster* in the NCBI BLASTp search were retrieved and aligned using the constraint-based multiple alignment tool (NCBI COBALT) (https://www.ncbi.nlm.nih.gov/tools/cobalt/re_cobalt.cgi/, accessed on 15 March 2021) with default parameters. The highly conserved and low-level conserved areas of each sequence were identified with different colors.

### 2.4. Evolutionary Analysis of Protein AA Sequences by Maximum Likelihood Method

Pelle, Tube, and Dorsal proteins from *D. melanogaster* and *D. citri* and insects belonging to several families that showed high similarity (over 25%) and had good coverage were selected for the analyzes. Multiple alignment of AA sequences was performed using CLUSTALW. The phylogenetic analysis of protein sequences was conducted using MEGA X software [64]. The evolutionary history of protein sequences for each protein was inferred using the maximum likelihood method and Kimura 2-parameter model for nucleotides [65], and the JTT matrix-based model for proteins [66]. Initial tree(s) for the heuristic search were obtained automatically by applying neighbor-joining and BioNJ algorithms to a matrix of pairwise distances estimated using the maximum composite likelihood (MCL) approach, and then selecting the topology with superior log likelihood value.

### 2.5. Generation of Three-Dimensional (3D) Structure Model

The protein structure homology modeling, structure-based function annotation, creation of three-dimensional (3D) structure, and prediction of the membrane topology of the predicted sequence were conducted using Swiss-model web software (https://swissmodel.expasy.org/, accessed on 14 April 2021). The PDB structure of each protein was generated by submitting the protein FASTA sequences to the website. The template was selected based on quality of the resulting models, as estimated by quaternary structure quality estimate (QSQE), global model quality estimate (GMQE), coverage, sequence identity, and sequence similarity [67]. The UCSF Chimera version 1.15 (https://www.cgl.ucsf.edu/chimera/, accessed on 15 May 2021) [68] was used to visualize binding site and protein modeling prediction.

### 2.6. Gene Expression Analysis Using Quantitative Real-Time PCR (RT-PCR)

For the gene expression analysis, we collected *D. citri* eggs and stages, including five nymphal instars (1st to 5th), teneral, and mature adults. Teneral is the newly emerged adult, it is pale and soft-bodied, and it lasts a few hours before it gets harder and darker (mature). Eggs were collected using forceps and nymphs were transferred to petri dishes using a fine camel hairbrush. Nymphs were examined under a stereoscope to classify them into instars based on size and morphological features. Teneral and mature adults were collected using an insect aspirator and sex was determined under the stereoscope. Total RNAs were extracted from different stages of *D. citri* using the RNase mini kit (Qiagen, Valencia, CA, USA). The quality and quantity of extracted RNA was measured by the Nanodrop 1000 spectrophotometer (ThermoFisher, Waltham, MA, USA). The gene expression was measured by quantitative RT-PCR as described by Nehela et al. (2018) [69]. The housekeeping genes actin, and *alpha-tubulin* were used as internal control genes [70] for transcript normalization because they showed high stability in *D. citri* under biotic stresses. Both internal control genes gave very similar results, however, we used *actin* for normalization. The gene expression for each biological sample was performed in triplicate. Primers for the Toll signaling pathway-related genes (Table 1) were used to perform the PCR. The 2−∆∆^C^_T_ method was used to measure the relative expression of consensus sequence among PCR products [71].

### 2.7. Statistical Analysis

The analysis of variance technique (ANOVA) was used for statistical comparison of the gene expression among different *D. citri* stages, followed by post hoc pairwise comparisons using the Tukey–Kramer honestly significant difference test (Tukey HSD; *p* < 0.05). Simple linear regression (SLR) analysis was performed to model the gene expression (as a dependent variable). The fitted regression line is expressed as a significant equation, as determined by the F test (*p* < 0.05). Both coefficients of determination (R2) and adjusted coefficient of determination (R2adj) were also obtained. Further, due to the observed nonlinear phenomena, data were fitted with a second-degree polynomial regression model (quadratic model) to understand the curvilinear relationship between life stage (as an independent variable) and gene expression (as a dependent variable). Polynomial regression models, the 95% confident curves for the estimated regression, quadratic equation, R2, R2adj, and *p*-value based on the F test (*p* < 0.05) were also obtained. JMP statistical software (SAS Institute, Cary, NC, USA) was used for all statistical analyzes listed above.

## 3. Results

### 3.1. In Silico Identification and Toll Pathway-Related Genes in D. citri

The protein–protein BLAST (BLASTp) search resulted in the identification of Toll-signaling pathway proteins, including Cactus, Toll, Pelle, Tube, Dorsal, Dorsal- immunity-related factor based on the NCBI database, while Spaetzle and MyD88 were identified based on the *D. citri* OGS v2 protein dataset website (https://citrusgreening.org/, accessed on 15 March 2021) because we did not find any similarity in the BLASTp search using NCBI (Table 2). Interestingly, the protein sequence of Dorsal and Dorsal immunity related factor from *D. melanogaster* showed significant similarity to same protein, the embryonic polarity protein Dorsal, such as isoform X1, X2, and X3. Among the Toll signaling pathway-related proteins, Tube, Pelle, and Dorsal were selected for further in silico analysis as described below.

### 3.2. Characterization of Diaphorina citri Pelle Protein (DcPelle)

**Figure 1:**
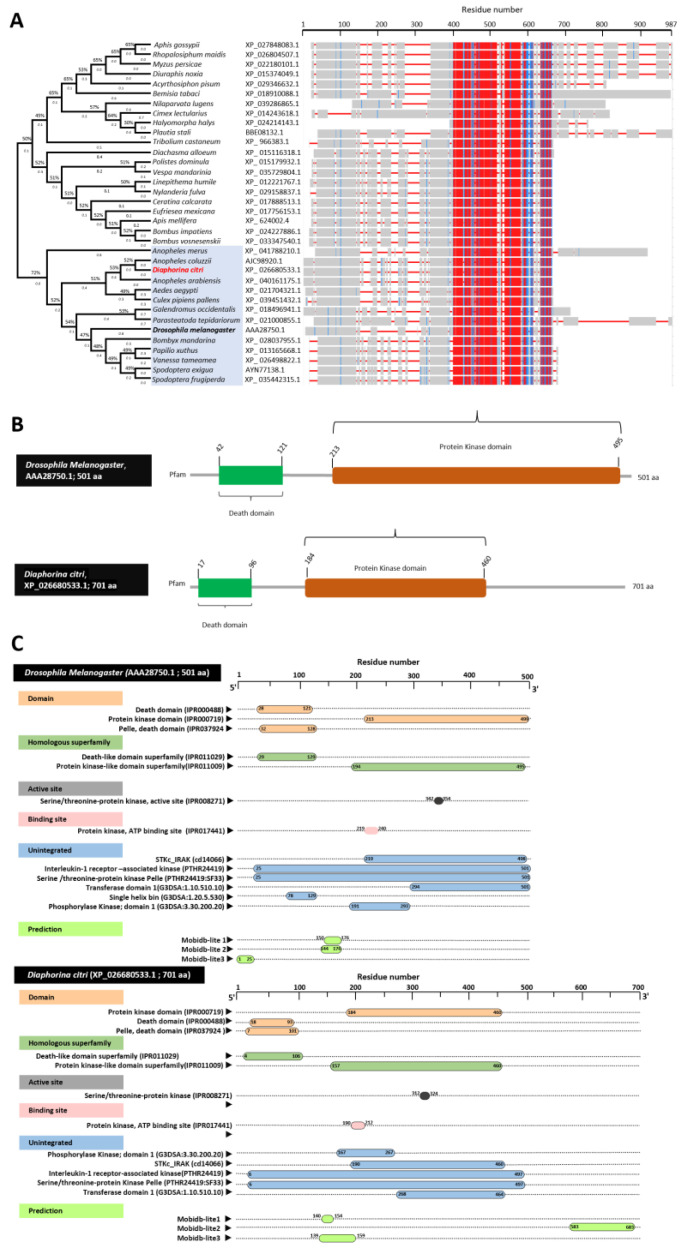
**Characterization Pelle protein of *D. citri* (DcPelle).** (**A**) Phylogenetic tree inferred from the protein sequences of the Pelle protein of *D. citri* and 34 other insect species. Evolutionary analysis carried out using the maximum likelihood method and JTT matrix-based model and bootstrap replicated 500 times. The protein sequences from *Pelle* gene were identified using protein–protein BLAST (BLASTp) using *Pelle* gene (*Pll*, AAA28750.1) from *D. melanogaster*, as a query sequence against *D. citri* and other insects available in GeneBank, the National Center for Biotechnology Information website (NCBI, https://www.ncbi.nlm.nih.gov/gene/, 15 April 2021). The phylogenetic tree was built from a protein sequence aligned by ClustalW and analyzed with MEGAX. The phylogenetic analysis involved 35 protein sequences. The branch lengths next to the branches show genetic changes and i.e., the longer the branch, the more genetic change. Branch length is measured in the number of substitutions per site. In COBALT, highest amino acid similarities/highest conserved sequence is presented in red, partial conservation/less conserved sequences are represented in blue, and the non-conserved area is in grey. (**B**) The profile and functional motif analysis of protein sequence in *D. citri* (XP_026680533.1) and *D. melanogaster* (AAA28750.1) using the ScanProsite online tool (https://www.expasy.org/resources/prosite/, accessed on 15 June 2021). (**C**) Domain and protein functional annotation of *D. melanogaster* (accession no. AAA28750.1) and *D. citri* (XP_026680533.1) using the InterPro Scan online tool (https://www.ebi.ac.uk/interpro/search/sequence/, accessed on 15 June 2021).

DmPelle (AAA28750.1) was aligned with the sequences of the top matched protein from the *D. citri* (DcPelle, XP_026680533.1) and other insect families. The multiple protein sequence alignment using ClustalW and phylogenetic trees analysis showed that DcPelle was phylogenetically closer (72%) to the query sequence DmPelle and Pelle in other insects (Figure 1A). Furthermore, the multiple AA alignment using COBALT set to the default parameters showed that DcPelle had high homology to the Pelle protein from other insects. Due to the difference in protein size in some insects, the lack of homology at the C-terminal ends did not make up the scoring homologous sequence. In addition, we found that about 200 amino acids were conserved among *D. citri* and other analyzed insects. The 200 conserved amino acid sequences in most insects (21 out of 35 insects in this study) begin with glutamine and end with aspartate (in 34 out of 35 insects). ScanProsite server predicted the presence of different functional motifs in Pelle protein in all insects, including the presence of protein kinase domain and conserved protein sequence (200 AA) in the protein kinase domain (Figure 1B). InterPro annotation of DcPelle and DmPelle resulted in three different assigned domains, homologous superfamilies, active sites, binding sites, and unintegrated proteins (Figure 1C). The conserved shared domains of the protein kinase domain (IPR000719), Pelle_death domain (IPR037924), and death domain (IPR000488) indicates high topological similarity between *D. citri* and *D. melanogaster* (Figure 1C). In brief, protein sequences of DcPelle and DmPelle have the protein kinase domain, pelle_death domain, death domain, death-like domain superfamily (DEATH-like_dom_sf, IPR011029), protein kinase-like domain superfamily (kinase-like_dom_sf, IPR011009), serine/threonine protein kinase (active site, IPR008271), protein kinase, ATP binding site (IPR017441), interleukin-1 receptor-associated kinase (PTHR24419), serine/threonine protein kinase Pelle (PTHR24419:SF33), transferase (phosphotransferase) domain 1 (G3DSA:1.10.510.10), phosphorylase kinase; domain 1 (G3DSA:3.30.200.20), catalytic domain of the serine/threonine kinases, interleukin-1 receptor-associated kinases and related STKs (STKc_IRAK, cd14066) (Figure 1C).

**Figure 2:**
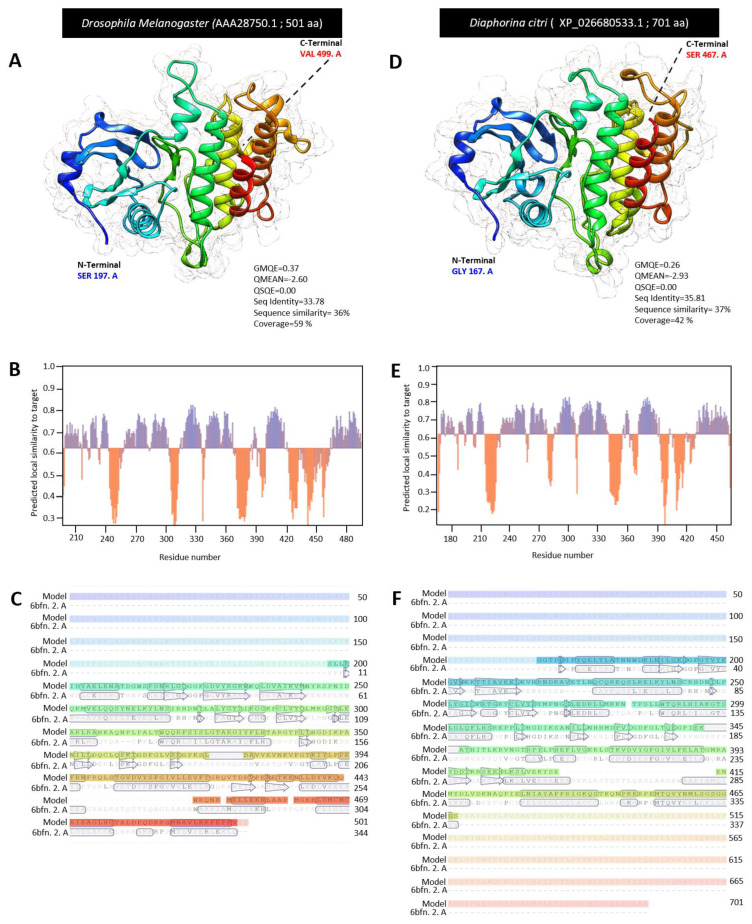
**The crystallographic three-dimensional (3D) modeling of Pelle protein of *D. citri.*** (**A**,**D**) The predicted three-dimensional (3D) structure model and its associated mesh surface of DmPelle (AAA28750.1), DcPelle (XP_026680533.1), respectively. The tertiary structures were predicted with the common template of crystal structure of human interleukin-1 receptor-associated kinase1 (also known as the crystal structure of human IRAK1) in the Protein Data Bank (PDB ID: 6bfn.2. A) between *D. citri* and *D. melanogaster* with 2.26 Å resolution. Protein chains are colored according to the rainbow color spectrum from blue (N-terminal) to the red (C-terminal). (**B**,**E**) Model–template alignment of DmPelle and DcPelle, respectively. Amino acid sequences of each model were aligned with template 6bfn.2. A. Secondary structure of each model are presented by α-helices (rectangles) and β-pleated sheets (arrows). Matched sequences in alignment are presented in black. (**C**,**F**) Local quality estimate of the predicted models of DmPelle and DcPelle, respectively. All bioinformatic analyzes were accomplished based on the most recent data available in GenBank, the National Center for Biotechnology Information website (NCBI, http://www.ncbi.nlm.nih.gov/gene/, accessed on 12 April 2021). The 3D structure of protein was generated using the Swiss-model server (https://swissmodel.expasy.org/, accessed on 12 July 2021) and visualized with UCSF Chimera package (version 1.15) (https://www.cgl.ucsf.edu/chimera/, accessed on 12 July 2021). QSQE: Quaternary structure quality estimate, GMQE: Global model quality estimation, and QMEAN z-scores: qualitative model energy analysis.

**Figure 3:**
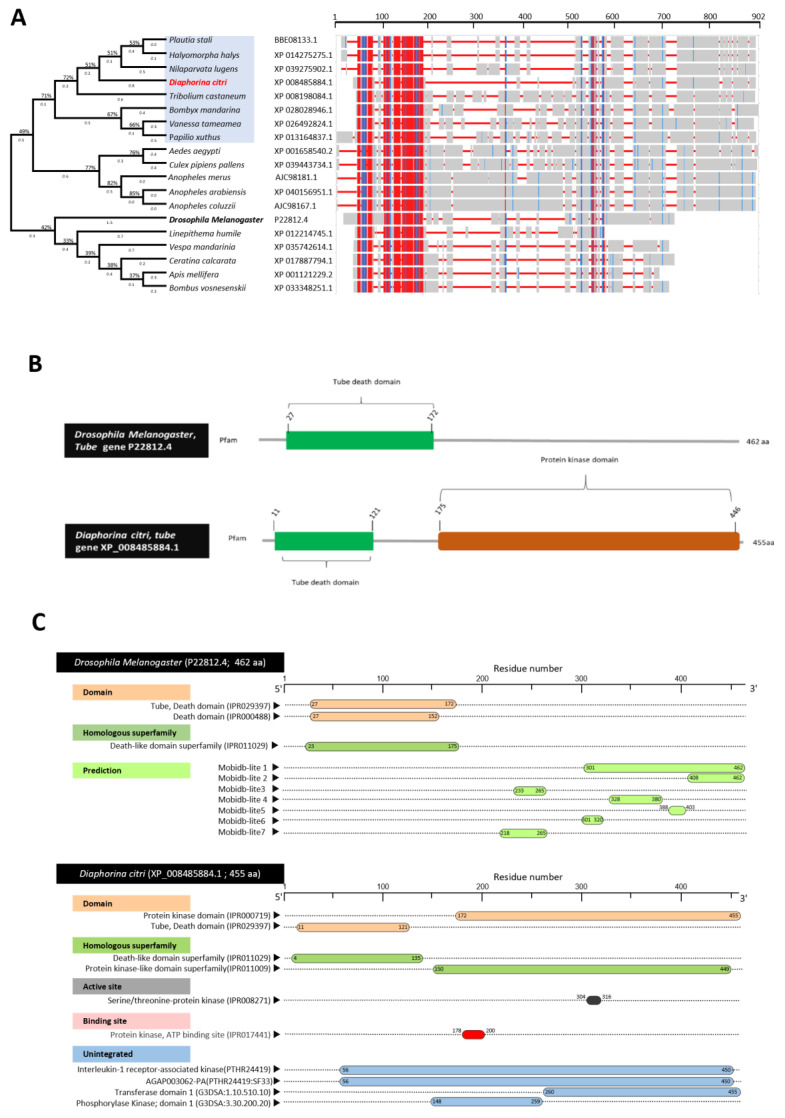
**Characterization Tube protein of *D. citri* (DcTube).** (**A**) Phylogenetic tree inferred from Tube protein sequence from *Diaphorina citri* and 18 other insect species. Evolutionary analysis carried out using the maximum likelihood method and JTT matrix-based model and bootstrap replicated 500 times. The protein sequences were identified using protein–protein BLAST (BLASTp) using *Tube* gene (*Tub* gene, GenBank accession no. P22812.4) from *D. melanogaster*, as query sequence against *D. citri* and other insects available in GeneBank, the National Center for Biotechnology Information website (NCBI, https://www.ncbi.nlm.nih.gov/gene/, accessed on 15 April 2021). The phylogenetic tree was built from a protein sequence aligned by ClustalW and analyzed with MEGAX. The phylogenetic analysis involved 19 protein sequences. The branch lengths next to the branches show genetic changes with the longer branches representing more genetic changes. Branch length is measured in the number of substitutions per site. In COBALT, the highest amino acid similarities/highest conserved sequence is presented in red, partial conservation/less conserved sequences are represented in blue, and non-conserved areas are in grey. (**B**) The profile and functional motif analysis of protein sequence in *D. citri* (accession no. XP_008485884.1) and *D. melanogaster* (accession no. P22812.4) using HMMER online tool (https://www.ebi.ac.uk/Tools/hmmer/, accessed on 6 July 2021) (**C**) Domain and protein functional annotation of *D. melanogaster* (accession no. P22812.4) and *D. citri* (accession no. XP_008485884.1) using the InterPro Scan online tool (https://www.ebi.ac.uk/interpro/search/sequence/, accessed on 6 July 2021).

For the homology modeling, we used Swiss modeling to compare the crystallographic three-dimensional (3D) structure of DcPelle, (XP_026680533.1) and DmPelle (AAA28750.1). The 3D structures of DcPelle and DmPelle were predicted using the crystal structure of human IRAK1 (also known as interleukin-1 receptor-associated kinase 1) in the Protein Data Bank (PDB ID: 6bfn.2. A), as the template for structural modeling. Based on the target and template chain sequence alignment, about 64% of amino acid residues of DmPelle (SER 197 to VAL 499) were modeled with template protein (seq identity = 33. 78%, sequence similarity = 36%, QSQE = 0.0, and coverage 59%) with reliable global model quality estimation (GMQE = 0.30) and good qualitative model energy analysis (QMEAN z-score = −2.60 (Figure 2A). The predicted model of DmPelle is a monomer with 15 α-helices and 14 β-sheets (Figure 2A,B) with considerable predicted local similarity to the target protein (Figure 3C). The predicted model does not have any ligand binding site, although the template has a ligand binding site for N-[2-methoxy-4-(morpholin-4-yl) phenyl]-6-(1H-pyrazol-5-yl) pyridine-2-carboxamide (DL1; residues Ile30, Phe 35, Val 38, Ala 49, Lys 51, Val 84, Tyr 100, Gly 101, Phe 102, Leu 103, Pro 104, Gly 106, Leu 159, and Asp 170).

Similarly, approximately 43% of amino acid residues (residues GLY 167 to SER 467) of DcPelle were modeled with the target protein (seq identity = 35.81%, sequence similarity = 37%, QSQE = 0.0, and coverage 42%) with good GMQE of 0.2 and reliable QMEAN of −2.93 (Figure 2D). The predicted model of DcPelle is a monomer consisting of 15 α-helices and 14 β-sheets (Figure 2D,E) with considerable predicted local similarity to the target (Figure 2F). Similar to the DmPelle, DcPelle is a monomer without ligand binding sites. Similarly, both predicted models of DmPelle and DcPelle have a similar number of α-helices and β-sheets (15 and 14, respectively).

### 3.3. Characterization of Diaphorina citri Tube Protein (DcTube)

DmTube (P22812.4) was aligned with the sequences of the top-matched protein sequences from the *D. citri* (DcTube, XP-008485884.1) and 17 other insects (Figure 3A). Multiple AA alignment sequences and phylogenetic trees showed that DcTube is clustered closely (approximately 72%) with the Tube proteins from four insects, including *Plautia stali* (BBE08133.1), *Halyomorpha halys* (XP_014275275.1), *Tribolium castaneum* (XP_008198084.1), and *Nilaparvata lugens* (XP_039275902.1). However, the Tube protein from *D. citri* was phylogenetically far from the DmTube query, with less than 50% similarity indicating diversity of Tube between the insect model and *D. citri*. Multiple protein sequence alignment using COBALT analysis showed that all predicted Tube protein sequences have high homology with the *D. citri* Tube protein (XP_008485884.1). COBALT analysis shows that around 150 amino acids were conserved among *D. citri* and the other insects in these analyzes (Figure 3A). HMMER analyzes of Tube sequences from all insects indicated that the death domain is present in all, and the protein kinases domain was only absent in a few insects. Interestingly, analyzing by COBALT and HMMER showed that the 150 AA-conserved amino acid sequences common among all insects were within the death domain, as it was a smaller region in the N-terminal.

The amino acid sequence analyzes of DcTube by InterPro Scan, showed that it has a protein kinase domain (IPR000719), Tube, death domain (IPR029397), death-like domain superfamily (IPR011029), protein kinase-like domain superfamily (IPR011009), serine/threonine-protein kinase (IPR008271) as the active site, protein kinase, ATP binding site (IPR017441), and unintegrated proteins, such as Interleukin-1 receptor-associated kinase (PTHR24419), AGAP003062-PA (PTHR24419:SF23), transferase domain 1 (G3DSA:1.10.510.10), phosphorylase kinase; domain 1 (G3DSA:3.30.200.20) and *D. melanogaster* having death domain (IPR000488), and death-like domain (IPR011029) as a superfamily (Figure 3B). Among the domains and superfamilies, the Tube death domain (IPR029397) was predicted as a conserved domain indicating high topological similarity between *D. citri* and *D. melanogaster* (Figure 3C).

**Figure 4:**
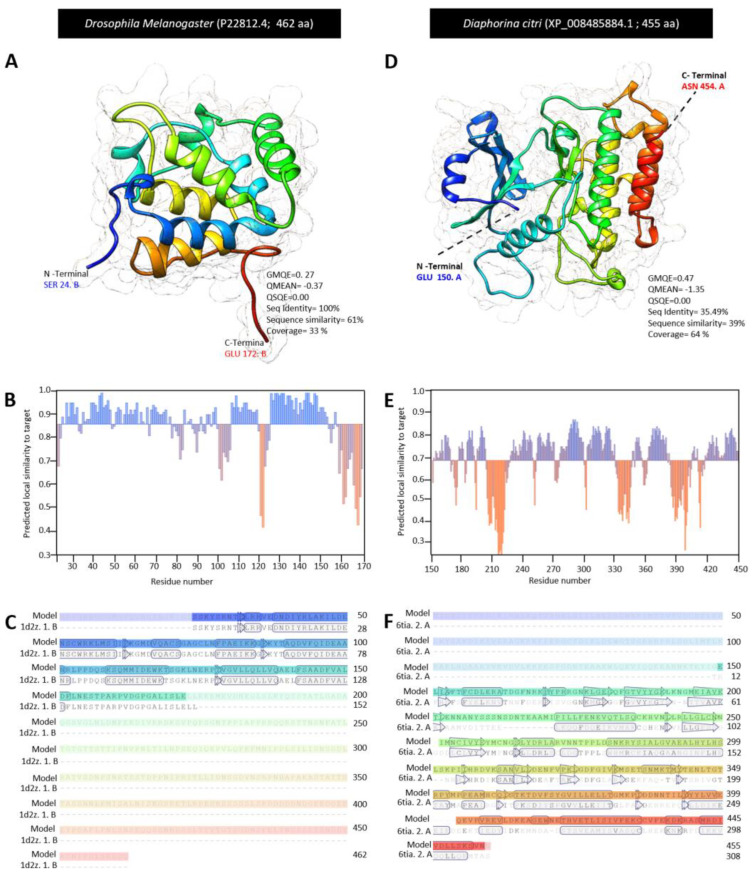
**The crystallographic three-dimensional (3D) modeling of Tube protein of *D. citri.*** (**A**,**D**) The predicted the three-dimensional (3D) structure model and its associated mesh surface of DmTube (P22812.4), and DcTube (XP_008485884.1), respectively. The tertiary structures of *D. melanogaster* and *D. citri* were predicted with the template of 3D structure of the complex between the death domains of Pelle and Tube (knows as the death domain of Tube) in the Protein Data Bank (PDP ID: 1d2z.1.B) with 2.00 Å resolution, and template of interleukin-1 receptor-associated kinase 4 (IRAK4 in complex with inhibitor) in the Protein Data Bank (PDP ID: 6tia.2.A) with 2.52 resolutions, respectively. Protein chains are colored according to the rainbow color spectrum from blue (N-terminal) to the red (C-terminal). (**B**,**E**) Model–template alignment of DmTube and DcTube, respectively. Amino acid sequences of *D. melanogaster* and *D. citri* template were aligned separately with 1d2z.1.B, and 6tia.2.A, respectively. The secondary structure of each model are presented by α-helices (rectangles) and β-pleated sheets (arrows). Matched sequences in alignment are presented in black. (**C**,**F**) Local quality estimate of the predicted models of DmTube, and DcTube, respectively. All bioinformatic analyzes were accomplished based on the most recent data available in GenBank, the National Center for Biotechnology Information website (NCBI, http://www.ncbi.nlm.nih.gov/gene/, accessed on 12 April 2021). The 3D structure of the protein was generated using the Swiss-model server (https://swissmodel.expasy.org/, accessed on 13 July 2021) and visualized with UCSF Chimera package (version 1.15) (https://www.cgl.ucsf.edu/chimera/, accessed on 13 July 2021). QSQE: quaternary structure quality estimate, GMQE: global model quality estimation, and QMEAN z-scores: qualitative model energy analysis.

The three-dimensional (3D) structure for DcTube was predicted using the Swiss-model server. Among all templates predicted by the Swiss-model, there was not a common template for DcTube and DmTube. Therefore, the most accurate template based on sequence identity and QMEAN z-scores was selected for *DmTube* and *DcTube* separately. Three-dimensional structure of the complex between the death domains of Pelle and Tube (death domain of Tube) with Protein Bank Data ID: 1d2z.1.B and 2.00 Å resolution was selected as template for structural modeling of DmTube. The 3D structure for the DcTube protein was built after alignment of the target sequence and the template structure of interleukin-1 receptor-associated kinase 4 (IRAK4 in complex with inhibitor) with PDP ID: 6tia. 2. A and 2.52 Å regulations. In DmTube, approximately 32% of amino acid residues (SER 24 to GLU 172) were modeled with template protein (seq identity = 100%, sequence similarity = 61%, QSQE = 0.0, and coverage 33%). The predicted protein model had a reliable global model quality estimation (GMQE = 0.22) and good qualitative model energy analysis (QMEAN Z -score = −0.37) (Figure 4A). The predicted model of DmTube is a monomer lacking the ligand binding site. It contains the eight α-helices and four β-sheets (Figure 4A,B) with considerable predicted local similarity to the target protein (Figure 4C).

In the predicted monomer DcTube, 67% of amino acid residues (residues GLU 150 to ASN 454) were modeled with the template protein (seq identity = 35.49%, sequence similarity = 39%, QSQE = 0.0, and coverage 64%) with a GMQE of 0.42 and an acceptable QMEAN score of −1.35 (Figure 4D). In the predicted DcTube model, 16 α-helices and 17 β-sheets are present (Figure 4D,E) with considerable predicted local similarity to the target (Figure 4F).

### 3.4. Characterization of Diaphorina citri Dorsal Protein (DcDorsal)

**Figure 5:**
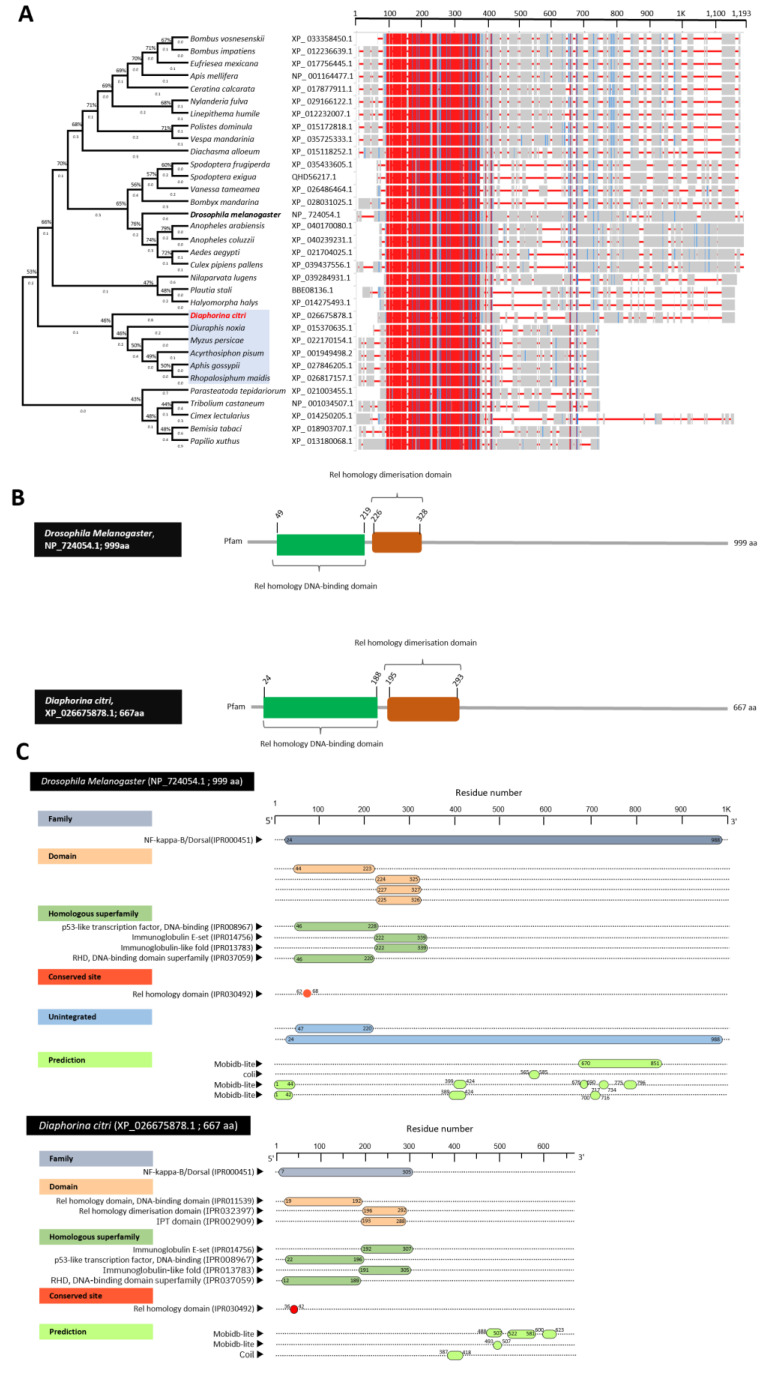
**Characterization Dorsal protein of *D. citri* (DcDorsal).** (**A**) Phylogenetic tree inferred from Dorsal protein sequence from *D. citri* and 32 other insect species. Phylogenetic tree inferred from Dorsal protein sequences of *D. citri* and other insects. Evolutionary analysis carried out using the maximum likelihood method and JTT matrix-based model, and bootstrap replicated 500 times. The protein sequences were identified using protein–protein BLAST (BLASTp) using a *dorsal* gene (*di* gene, NP_724054.1) from *D. melanogaster*, as a query sequence against *D. citri* and other insects available in GeneBank, the National Center for Biotechnology Information website (NCBI, https://www.ncbi.nlm.nih.gov/gene/, accessed on 15 April 2021). The phylogenetic tree was built from protein sequences aligned by ClustalW and analyzed with MEGAX. The phylogenetic analysis involved 33 protein sequences. The branch lengths next to the branches show genetic changes with longer branches inferring more genetic change. Branch length is measured in the number of substitutions per site. In COBALT, highest amino acid similarities/highest conserved sequence is presented in red, partial conservation/less conserved sequence is represented in blue, and non-conserved area is in grey. (**B**)The profile and functional motif analysis of the protein sequence in *D. citri* (accession no. XP_026675878.1) and *D. melanogaster* (accession no. NP_724054.1) using HMMER (https://www.ebi.ac.uk/Tools/hmmer/, accessed on 12 July 2021). (**C**) Domain and protein functional annotation of *D. melanogaster* (NP_724054.1) and *D. citri* (XP_026675878.1) using the InterPro Scan online tool (https://www.ebi.ac.uk/interpro/search/sequence/, accessed on 15 June 2021).

The BLASTp search of DmDorsal (NP_724054.1) as the query in NCBI resulted in the identification of the Dorsal protein in *D. citri*. The BLASTp search of *D. melanogaster* produced significant similarities to the embryonic polarity protein Dorsal-like isoform X1 (XP_017305228.1), embryonic polarity protein Dorsal-like isoform X2 (XP_026675878.1) and embryonic polarity protein Dorsal-like isoform X3 (XP_017305229.1). The availability of Dorsal protein sequences from different insect families allowed us to refine the phylogenetic analysis of Dorsal protein sequences across species. Multiple alignment of top-matched amino acid sequence in BLASTp search using ClustalW resulted in the construction of a phylogenetic tree (Figure 5A). The phylogenetic analysis reveals that Dorsal proteins from the insects analyzed fell in different clades, showing that they share a common ancestor. DcDorsal (accession no. XP_026675878.1) is closely (approximately 46%) related to the aphids *Diuraphis noxia* (XP_015370635.1), *Myzus persicae* (XP_022170154.1), *Acyrthosiphon pisum* (XP_001949498.2), *Aphis gossypii* (XP_027846205.1), and *Rhopalosiphum maidis* (XP_026817157.1) belonging to the Hemiptera. However, DcDorsal and DmDorsal fell into two different clades, showing divergence in Dorsal protein between these two insects (Figure 5A). Multiple amino acid sequence alignment using COBALT showed about 300AA of the Dorsal protein sequence is highly similar and highly conserved (Figure 5A). Furthermore, the HMMER search for novel domains with standard parameters was performed against the protein family database (Pfam) for potential domain matches. HMMER search revealed two potential domains of Rel homology DNA-binding domain and Rel homology dimerization domain in all insects. Analysis by COBALT and HMMER showed amino acid (300 AA red area in COBALT) thought Rel homology DNA-binding and Rel homology dimerization domains are highly conserved in all insects (300 conserved amino acids in these two domains in all insects) (Figure 5B).

Annotation of the Dorsal protein sequence using InterPro scan in DcDorsal (NP_724054.1) and DmDorsal (XP_026675878.1) showed that they share NF-kappa-B/Dorsal (IPR000451), Rel homology domain (RHD), DNA-binding domain (IPR011539), IPT domain (IPR002909), Rel homology dimerization domain (IPR032397), p53-like transcription factor, DNA-binding (IPR008967), immunoglobulin E-set (IPR014756), immunoglobulin-like fold (IPR013783), DNA-binding domain superfamily (IPR037059), and Rel homology domain as a conserved site (IPR030492) (Figure 5C). Although unintegrated proteins, such as N-terminalsub-domain of the RHD of the arthropod protein Dorsal (cd07887) and Dorsal-related immunity factor DIF-related (PTHR24169:SF20) were missing in DcDorsal but present in DmDorsal. The Dorsal protein annotation indicated that most of the domain, family protein, homologous superfamily, and conserved site is conserved between them.

**Figure 6:**
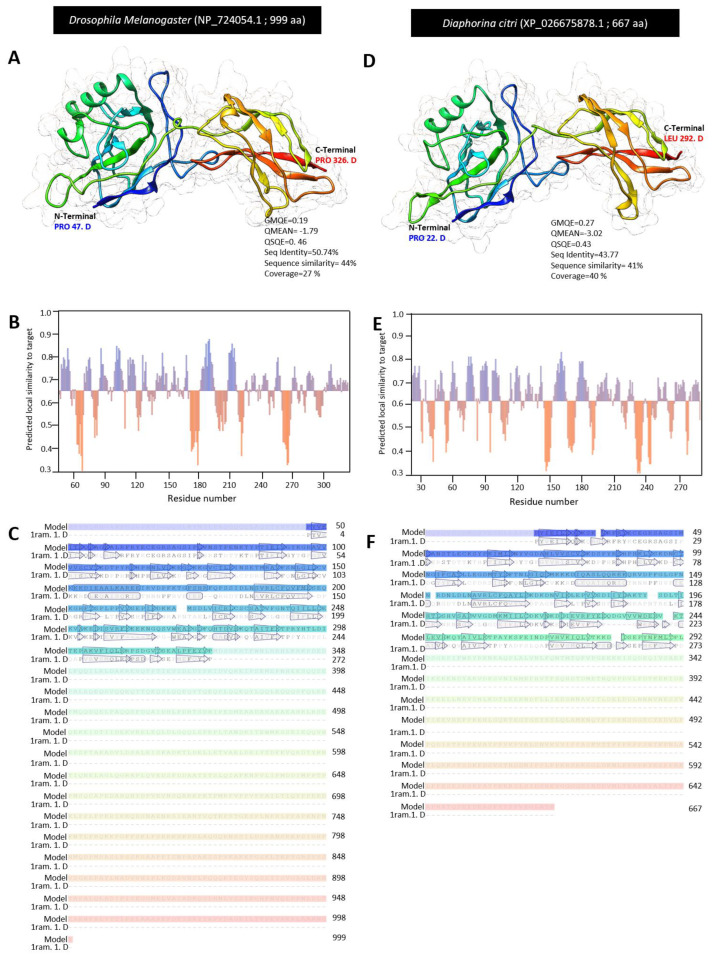
**The crystallographic three-dimensional (3D) modeling of Dorsal protein of *D. citri.*** (**A**,**D**) The predicted three-dimensional (3D) structure model and its associated mesh surface of DmDorsal (NP_724054.1) and DcDorsal (XP_026675878.1), respectively. The tertiary structures of *Drosophila melanogaster* and *D. citri* were predicted with the template of a 3D structure protein (transcription factor NF-κBp65) as a novel DNA recognition mode by NF-κB P65 homodimer in the Protein Data Bank (PDP ID: 1ram.1.D with 2.70 Å resolution). Protein chains are colored according to the rainbow color spectrum from blue (N-terminal) to red (C-terminal). (**B**,**E**) Model–template alignment of DmDorsal and DcDorsal, respectively. Amino acid sequences of each model were aligned with template 1ram.1.D. The secondary structures of each model are presented by α-helices (rectangles) and β-pleated sheets (arrows). Matched sequences in alignment are presented in black. (**C**,**F**) Local quality estimate of the predicted models of DmDorsal and DcDorsal, respectively. All bioinformatic analyzes were accomplished based on the most recent data available in GenBank, the National Center for Biotechnology Information website (NCBI, http://www.ncbi.nlm.nih.gov/gene/, accessed on 12 April 2021). The protein 3D structure was generated using the Swiss-model server (https://swissmodel.expasy.org/, accessed on 13 July 2021) and visualized with the UCSF Chimera package (version 1.15) (https://www.cgl.ucsf.edu/chimera/, accessed on 12 July 2021). QSQE: quaternary structure quality estimate, GMQE: Global model quality estimation, and QMEAN z-scores: qualitative model energy analysis.

The crystallographic three-dimensional (3D) structure of DcDorsal and DmDorsal was predicted using Swiss modling. The 3D structure of DmDorsal and DcDorsal was predicted using the protein transcription factor NF-κB P65 (a novel DNA recognition mode by NF-κB P65 homodimer) in the Protein Data Bank (PDB ID: 1ram.1. D) as the template for structural modeling. Based on target and template chain sequence alignment, about 28% of amino acid residues of DmDorsal (PRO 47 to PRO 326) were modeled with the template protein (PDB ID: 1ram.1. D) (seq identity = 50.74%, sequence similarity = 44% QSQE = 0.45, and coverage 27%) with valid global model quality estimation (GMQE = 0.16) and good qualitative model energy analysis (QMEAN z-score = −1.79 (Figure 6A). The predicted model for DmDorsal is a monomer, with the lack of a ligand binding site and it contains three α-helices and 29 β-sheets (Figure 6A,B) with considerable predicted local similarity to the target protein (Figure 6C).

Likewise, approximately 40% of amino acid residues (residues PRO 22 to LEU 292) of DcDorsal were modeled with the target protein (PDB ID: 1ram.1. D) (seq identity = 43.77%, sequence similarity = 41%, QSQE = 0.43, and coverage 40%) with good GMQE of 0.23 and reliable QMEAN of −3.02 (Figure 6D). Similar to DmDorsal, the 3D structure of DcDorsal was monomeric with no ligand binding site and it contains 1 α-helix and 29 β-sheets (Figure 6D,E), with reasonable predicted local similarity to the target (Figure 6F). The predicted 3D structures of the Dorsal protein in both DmDorsal and DcDorsal were similar (Figure 6A,D).

### 3.5. Expression of Toll Pathway-Related Genes at Different Developmental Stage of D. citri

**Figure 7:**
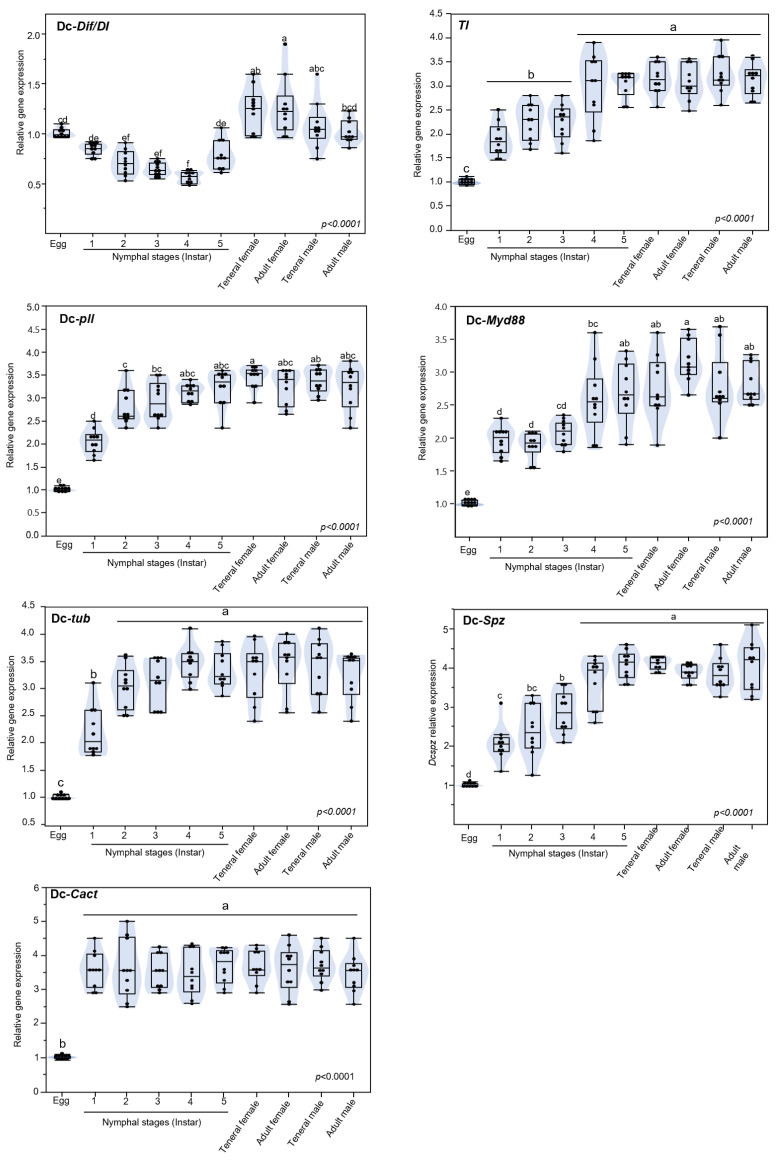
**The relative expression level of genes involved in Toll pathway in *D. citri***. Dorsal related immunity factor/Dorsal (*Dif/DI*), Pelle (*pll*), Tube (*tub*), Cactus (*cact*), Toll (*TI*), Myeloid differentiation factor 88 (*Myd88*), and Spaetzle (*spz*) genes expression in different developmental stages of *D. citri.* Expression of each gene is presented with an overlapping boxplot, violin plot, and scatter plot. In the boxplot, whiskers represent the maximum and minimum values, horizontal thick lines in the middle of the box shows the median, and boxes show the middle 50% of scores (i.e., the range between the 25th and 75th percentile, interquartile ranges (IQR)). Differences between developmental stages were statistically evaluated for each gene by the one-way ANOVA (analysis of variance) with Turkey HSD (*p* < 0.05).

The level of transcripts of the seven Toll pathway-related genes *Dif/DI*, *Pll*, *tub*, *cact*, *TI*, *Myd88*, and *spz* were analyzed at eight developmental stages of *D. citri* eggs, first, second, third, fourth, and fifth nymphal instars and teneral and mature adults, both females and males. *DcDif*/*DI* genes were found to be expressed throughout developmental stages, with the strongest level of expression in eggs and teneral, as well as mature adults both male and female (Figure 7). The six remaining Toll pathway-related genes followed a similar expression pattern of upregulation during development from the egg to the adult stage, with slight fluctuation at all eight developmental stages. However, the lowest level of expression was observed for eggs. In *tub*, *pll,* and *myd88* genes, adult females had the highest expression while adult males expressed *spz* gene the most. In the *cact* and *TI* gene, the second and fourth nymph instar had the highest expression, respectively.

**Figure 8:**
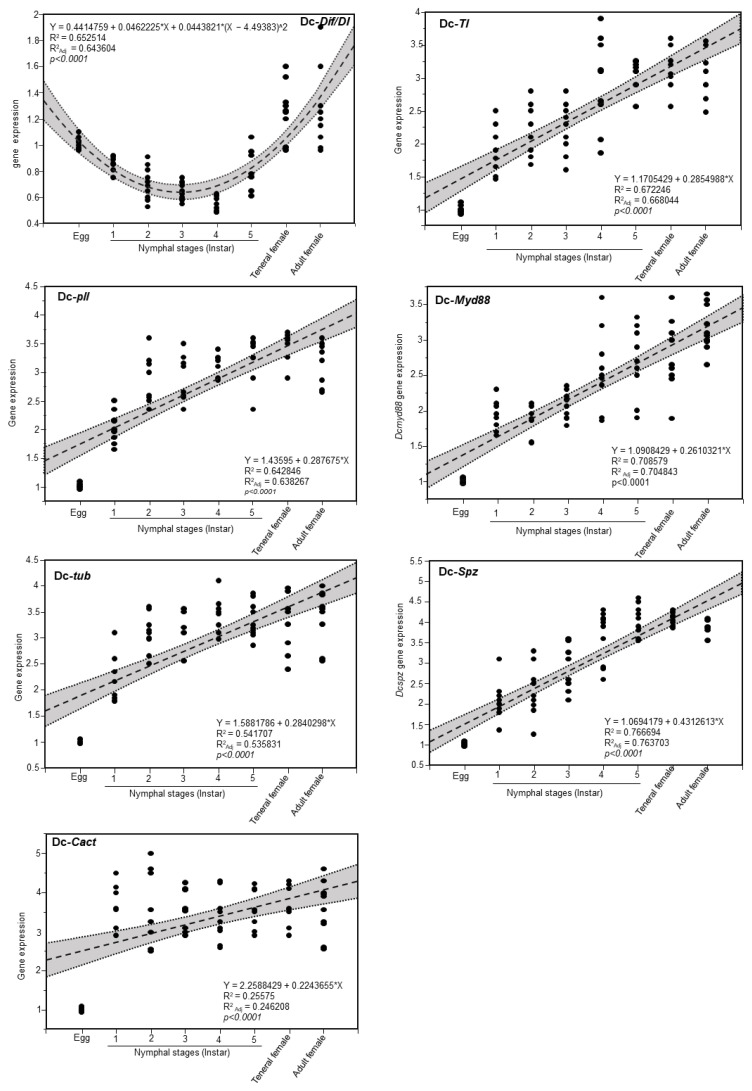
**Regression of the relative expression level of genes involved in the Toll pathway in *D. citri***. Dorsal-related immunity factor/Dorsal (Dif/DI), Pelle (pll), Tube (tub), Cactus (cact), Toll (TI), Myeloid differentiation factor 88 (Myd88), and Spaetzle (spz) genes expression in different developmental stages of *D. citri*. Quadratic polynomial regression analysis between Dif/DI gene expression and developmental stages of *D. citri*. Simple linear regression analysis between pll, tub, cact, TI, myd88, spz genes expression and *D. citri* developmental stages. In the linear and quadratic polynomial regression, dots show the raw data. The black round shapes indicate the raw data (*n* = 10). The fitted and polynomial regression line is presented as dashed lines. The 95% confidence interval (CI) for estimated regression is presented as gray-shaded and edged by dotted lines. The regression equation, RSquare (R^2^) and Rsquare Adj (R^2^adj), and *p*-value obtained after F tests are presented within each graph.

Because of the nonlinear relationship in the *DcDif*/*DI* gene expression in developmental stages, data were fitted using a second-degree polynomial regression model with significant differences in expression level (Figure 8). This polynomial regression confirms the implication of this gene in embryonic development. Data for the other six genes were fitted using a simple linear regression (SLR) with a *p*-value of less than 0.0001, indicating the immunity increased by the development and reached the maximum in adults (Figure 8).

## 4. Discussion

Three major signaling pathways are involved in the innate immune system of insects against pathogens, including Toll, IMD, and JAK/STAT [32]. The Toll pathway is activated by viruses, fungi, and Gram-positive bacteria [72,73] and IMD pathway is activated by Gram-negative bacteria [74], while the JAK/STAT pathway coordinates immune responses from cytokines and regulates multiple homeostasis mechanisms in the host [37]. *D. citri* has the Toll and JAK/STAT pathways but lacks the IMD pathway, suggesting that *D. citri* has a reduced immune system that enables *D. citri* to be infected with ‘*Ca.* L. asiaticus*’* [47,51]. However, there is a probability that *D. citri* uses the endosymbiont-resulting defense or the cellular immune defense, which may not be evident in the genome annotation [47,51].

The Toll pathway signaling is key for mediating innate immune responses and producing AMPs in some insect species [72]. This system was not well characterized in *D. citri.* Our comprehensive in silico and bioinformatic analyzes using protein–protein BLAST of *Drosophila melanogaster* as a model against *D. citri* in the NCBI and *Diaphorina citri* OGS v2.0 protein databases revealed seven Toll pathway-related genes in *D. citri. Cact*, *Pll*, *tub*, *TI*, *Dif*/*dI*, *Myd88*, and *spz* identified in *D. citri,* and they were characterized in silico by the analysis of domain composition, tertiary structure, and phylogeny. In addition, gene expression patterns in different life stages were performed with the goal of better understanding the biological roles of the Toll pathway in *D. citri*.

The Toll signaling system is required for embryonic Dorsal–ventral pattern formation, and for innate immunity against pathogens in adult insects. The Gram-positive bacterial or fungal infection activates the Toll pathway signaling and stimulates protease cascade enzymes, which finally cleave the Spaetzle protein of the host. This cleaved Spaetzle binds to one of the Toll receptors, and Spaetzle-mediated activation leads to the interaction of three cytoplasmic proteins, Pelle, Tube, and MYD88 via the death domain, underneath the cell membrane. Subsequently, the signaling complex of Pelle/Tube/MyD88 leads to the phosphorylation and proteosome degradation of cytoplasmic components of NF-κB proteins Dorsal/Dif and IκB protein Cactus. The degradation of IκB protein Cactus following the Toll pathway activation causes their release and the nuclear location of the Dif and Dorsal, and expression of antimicrobial peptides (AMPs) *Drosomycin* and *Immune induced molecule 1* (IM1) in the fat body in response to infection [38,60,75].

Spaetzle protein consists of a C-terminal and the pro-domain region that is synthesized and secreted as an inactive form of pro-protein and processed to the functional form of C-106 by serin protease cascades. Tube and MyD88 function as protein adaptors when the receptor is active. The TIR interleukin-1 receptor domain of Toll binds to the domain of MyD88, but Myd88 and Pelle do not contact each other directly; instead, two separate death domains on the surface of the adaptor protein, Tube, connect the MyD88 and Pelle. In fact, Tube, as an adaptor, engages the Pelle kinase at the formed complex with Toll and functions upstream of Pelle and downstream of Tube. Pelle is a serine/threonine kinase that contains the C-terminal catalytic domain, and also contains an N-terminal death domain. It is shown that Pelle, because of its kinase activity, causes autophosphorylation, induces phosphorylation, and subsequently degradation of the inhibitor Cactus in two separate N-terminal motifs [76,77]. Tube is an interleukin-1 receptor-associated kinase 4 (IRAK-4) homolog from the kinase protein family lacking catalytic domains. Tube contains an amino-terminal death domain and C-terminal region that participates in protein–protein interactions. The death domain of Tube plays a bridge role between death domains of Pelle and Myd88. Pelle, Tube, and IRAK have a common ancestral gene. In fact, the primary role of the Tube and Pelle death domain is to bring the Pelle kinase domain in indirect connection with Myd88 [78]. Cactus, in a non-signaling condition, is attached to the Dif and/or Dorsal and prevents nuclear localization and their activity, so Cactus degradation is needed for nuclear transaction for *Dif/di.* Upon the infection and signal induction, Cactus degradation occurs and causes the translocation of Dif/di to the nucleus, which activates the expression of antimicrobial peptides [79].

The Toll/Cactus signaling pathway plays an important role in regulating density and proliferation of hemocytes during larval development of *D. melanogaster* [80]. The NF-κB factors, Dorsal, and Dorsal-related immunity factor (DIF), are the most important humoral immune response regulators because they function with the production of antimicrobial peptides (AMPs) in Toll signaling. The DIF gene does not participate in Dorsal–ventral patterning during embryonic development. Instead, it cooperates with Cactus, and it engages in immune responses in larva and adult fruit flies. The Dorsal gene controls expression of zygotic genes, both of which are essential for Dorsal–ventral patterning in early embryonic development [81].

Based on the above, we focused on three main genes of Toll pathway signaling, including Pelle, Tube, and Dorsal for further analyzes in *D. citri*. Our InterPro-based analysis showed that the Pelle protein has kinase and death domains. The analyzes suggest that Pelle protein in both *D. citri* and *D. melanogaster* function similarly. The Tube protein in *D. citri* possesses kinase domain that is absent in *D. melanogaster* Tube protein, indicating that it is remarkably similar in overall structure to Pelle in *D. melanogaster* and IRAK4 in mammalian cells. In addition, the presence of a death domain in the Tube protein of *D. citri* and *D. melanogaster* does not require their association with a protein kinase family protein, as death domains are observed in many proteins lacking catalytic function or a protein kinase domain, and this result agrees with findings by Towb et al. [78]. The IRAK4 in mammals have a kinase domain, which is conserved highly in the catalytic domain, and functions by moderating the complex assembly in Toll pathway signaling [78]. Kinase proteins are divided into three classes: serine/threonine protein kinases, tyrosine protein kinases, and dual specificity protein kinases [82]. Our results agree with the findings in which Pelle and Tube are orthologous to IRAK1, and IRAK4 in mammals [78].

In our protein–protein BLAST search of the Dif/dI protein sequence from *D. melanogaster* against the *D. citri*, both genes hit the same protein of embryonic polarity protein Dorsal-like. *Dif*/*dI* and NF-κB are similar homologues that are translocated in fat body cell nuclei upon Toll pathway activation by pathogens, but they have distinct functions. *dI* plays an important role in early embryo development, while *Dif* is is involved in the adult immune system [83]. Our in silico analyzes showed that *Dif* genes are absent in *D. citri* and it have only a *Dorsal* gene (dI). This result is consistent with Berni et al. (2014) [84] in which the authors showed that Dorsal immune factor (dif) ortholog codes for the Rel-like protein in *D. melanogaster*, and is not present in the genome of *Rhodnius prolixus* and other insects.

In the current study, we investigated the expression dynamics of Toll pathway genes with the goal of better understanding their roles in the development and physiology of *D. citri* during all developmental stages. Our result show that *dI* is expressed higher in eggs and female teneral, as well as in adults, indicating that the female delivers the majority of *DcdI* mRNAs required for embryonic developmental. In fact, RNAi of *dI* in the early stages of *Rhodnius prolixus* decreased the mRNAs level up to 90% and caused misplaced anterior embryonic and severe defects in embryonic development, suggesting embryo patterning is controlled by *dI* maternally [84]. In addition, Bingsohn et al. (2017) [57] showed that RNAi knockdown of Pelle and Dorsal prevents the hatching of eggs in the next generation.

On the other hand, *Myd88*, *tub*, *pll*, *Ti,* and *Spz* genes followed similar expression patterns and were upregulated during developmental stages from eggs to the adult stages, and reached the maximum expression in late nymphal stages and adult. Our results show that *cact* was expressed at similar levels, but the lowest expression level was found in eggs. This result was in agreement with gene expression in *Tribolium castaneum,* in which most of the genes were upregulated during developmental stages from larva to adult and reached the highest expression in the pupal and adult stages [57]. In contrast to our results, in *T. castaneum cact*, *Dif*, *Myd88*, *tub*, and *pll* genes had the highest expression level in eggs compared to the developmental larval stages. Similar to our results, *cact* gene expression in *T. castaneum* was similar in all developmental stages but was lowest in the fifth instar.

## 5. Conclusions

**Figure 9:**
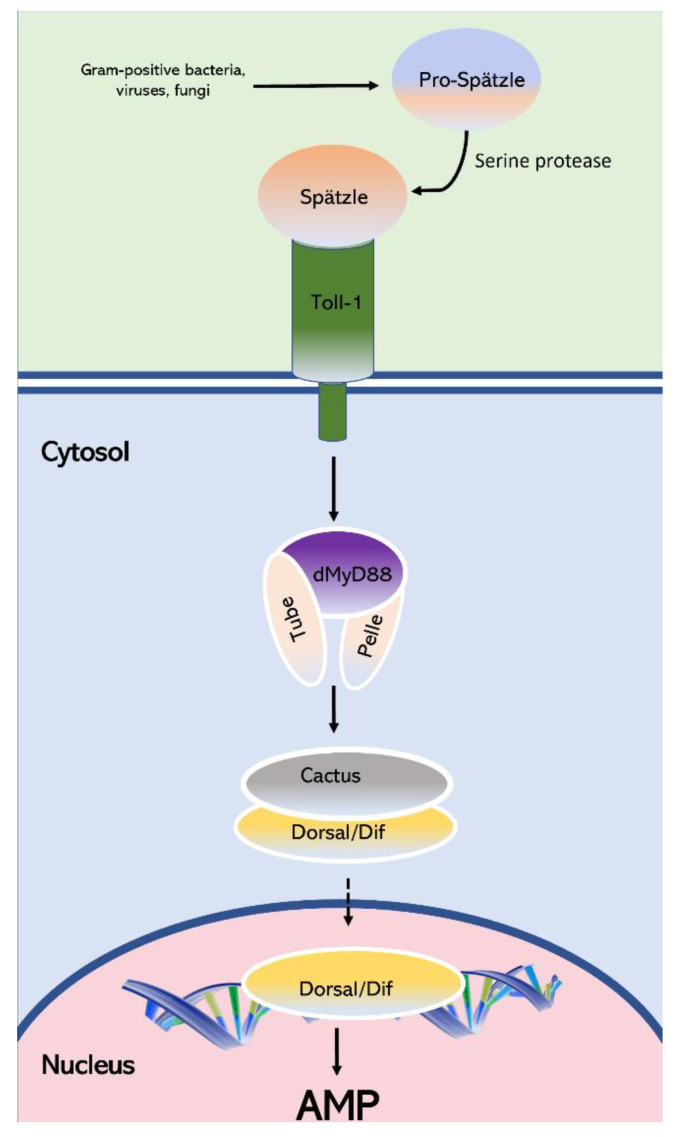
A schematic representation of Toll signaling system in *D. citri*. The model highlights the Toll signaling pathway as it functions during the immune response. The pattern recognition receptors recognize pathogens and activate a serin protease cascade and cause a binding of Speatzle to Toll. Toll binds to Myd88 through TIR domains and induces Myd88 to bind to Tube and Pelle through their death domains and make a triangle. The bindings lead to the recruitment of two proteins, the adaptor Tube and kinases Pelle. This results in the proteolytic degradation of the inhibitor kB (IkB) protein Cactus and activation of the NF-kB proteins Dif and Dorsal (same protein in *D. citri*). The activated Dorsal/Dif is translocated to the nucleus and induces the transcription and production of antimicrobial peptides (AMP).

To elucidate the mechanisms regulating the innate immunity in *D. citri*, we identified the Toll system signaling pathway-related genes. Comprehensive in silico analyzes and gene expression assays were performed. Our overall findings suggest that *D. citri* possesses an active Toll signaling pathway with the major proteins; however, some unique differences from most insects were found, such as the presence of a kinase domain in Pelle protein, and that the Dorsal immunity factor and Dorsal are most likely the same protein. The gene expression patterns indicate that the innate immunity increases with maturity of *D. citri,* as the higher expression of genes is in adults. Taking all these findings together, Figure 9 illustrates the suggested Toll signaling pathway in *D. citri*. This work opens a new avenue to designing innovative strategies for controlling *D. citri*. For example, silencing Toll signaling-related genes may increase *D. citri’s* susceptibility to pathogens that are used for biological control. Combining RNA interference or CRISPR with biological control applications could be an efficient control strategy for mitigating HLB.

## Figures and Tables

**Table 1:** List of primers used in this study for gene expression.

Gene	Primers	Nucleotide Sequence 5′-3′	Length (bp)
*Cact*	Dc-*Cact* F	GAGCTAGCACAAGTGATGGG	147
	Dc-*Cact* R	CAACACTAGCTGTCGCATGA	
*Dif*/*Di*	Dc-*Dif* F	AGCGTATCTGCCCAAGGATA	120
	Dc_*Dif* R	GCAGACACATGGGACAAAGT	
*TI*	Dc-*TI* F	TTGTCGGTATCCTGACCCTT	112
	Dc-*TI* R	CGATCGGTCCTAATCGGTTG	
*Myd88*	Dc-*Myd88* F	TTGAAGCCATCAGGCCAAAG	147
	Dc-*Myd88* R	CACAGTTGGGATTTGGAGGG	
*Spz*	Dc-*Spz* F	GGTGCGTCCAGGAATACATC	122
	Dc-*Spz* R	GGGTTCAAAGTTGTGTTGCG	
*pll*	Dc-*pll* F	CCAGAGCAGAGCTGTGTAGCA	161
	Dc-*pll* R	CTGAGCACCCCACTTGGACA	
*tub*	Dc-*tub* F	AACCGGAATCTCCCGGTGAC	143
	Dc-*Tub* R	ACCATCAGTAGCGCGTTCCA	
actin	Dc-*Actin* F	CCCTGGACTTTGAACAGGAA	170
	Dc-*Actin* R	CTCGTGGATACCGCAAGATT	
Alpha-tubulin	Dc-*Tubulin* F	CTTTCCAACACCACCGCTAT	142
	Dc-*Tubulin* R	GGTCTTCCCTCGCCTCTG	

**Table 2:** Alignment statistics for the top-matched sequences producing significant alignments of Toll system genes.

*Drosophila melanogaster* (Accession #)	*Diaphorina citri*							
	Annotation in *D. citri*	Accession #	aa	Max Score	Total Score	Query Cover %	E Value	Identity %
Cactus (AAA85908.1).	NF-kappa-B inhibitor Cactus	XP_008487783.1	304	122	122	34	1 × 10^−31^	37.57
* Dorsal related immunity factor (AAA28465.1)	embryonic polarity protein Dorsal-like isoform X3	XP_017305229.1	541	211	211	47	9 × 10^−61^	39.44
	embryonic polarity protein Dorsal-like isoform X2	XP_026675878.1	667	208	208	44	2 × 10^−58^	41.14
	embryonic polarity protein Dorsal-like isoform X1	XP_017305228.1	691	208	208	44	2 × 10^−58^	41.14
Toll (AAA28941.1)	protein Toll-like	XP_026684268.1	857	283	283	68	4 × 10^−81^	29.54
	protein Toll-like	XP_026685794.1	1109	269	403	76	4 × 10^−75^	30.8
	protein Toll-like	XP_008479067.1	169	63.2	63.2	11	2 × 10^−11^	24.81
Pelle (AAA28750.1)	serine/threonine-protein kinase pelle-like isoform X3	XP_026680533.1	701	266	266	90	2 × 10^−81^	38
	serine/threonine-protein kinase pelle-like isoform X2	XP_026680532.1	703	266	266	90	2 × 10^−81^	38
	serine/threonine-protein kinase pelle-like isoform X1	XP_026680531.1	708	266	266	90	2 × 10^−81^	38
** Myd88 (AAL56570.1)	myd88-RA. (AHRD V3.11 *** Myeloid differentiation primary response protein MyD88 tr	DcitrP049290.1.1	396	92.0			5 × 10^−20^	28.06
Tube (P22812.4)	protein Tube, partial	XP_008486711.1	229	67.0	67.0	33	8 × 10^−13^	29.49
	interleukin-1 receptor-associated kinase 4-like	XP_008485884.1	455	67.0	67.0	33	5 × 10^−12^	29.49
** Spaetzle (NP_733194.1)	spaetzle5-PA partial	DcitrP089585.1.5	296	-	-	-	2 × 10^−18^	30.61
	spaetzle5-PA partial	DcitrP089585.1.4	296	-	-	-	2 × 10^−18^	30.61
	spaetzle5-PA partial	DcitrP089585.1.3	296	-	-	-	2 × 10^−18^	30.61
	spaetzle5-PA partial	DcitrP089585.1.2	296	-	-	-	2 × 10^−18^	30.61
	spaetzle5-PA partial	DcitrP089585.1.1	296	-	-	-	2 × 10^−18^	30.61
* Dorsal (Di; NP_724054.1)	embryonic polarity protein Dorsal-like isoform X2	XP_026675878.1	667	289	384	52	1 × 10^−85^	47.38
	embryonic polarity protein Dorsal-like isoform X1	XP_017305228.1	691	289	384	53	2 × 10^−85^	46.85
	embryonic polarity protein Dorsal-like isoform X3	XP_017305229.1	541	280	280	32	7 × 10^−84^	46.58

* Dorsal immunity related factor and Dorsal of *Drosophila melanogaster* hit the same sequence from *D. citri* in blast search in NCBI. ** No significant similarity found in NCBI blast search for these two genes, so *D. citri* website (https://citrusgreening.org/organism/Diaphorina_citri/genome/, accessed on 15 March 2021) were used for BLASTp search. The remaining genes showed similarity in both NCBI and the *D. citri* website except the *tube* gene which did not show similarity in the *D. citri* website.

## Data Availability

Data will be shared upon request to the corresponding author.
